# 

**Published:** 1891-07

**Authors:** 


### CONTENTS:

Article I.—The Retention or Removal of the Sixth-Year Molar—Which? By Theodore F. Chupein. - - - 97

" II.—Discussion on Anæsthetics in the Therapeutic Section of the British Medical Association, at the Annual General Meeting at Bournemouth, 1891. - - 104

" III.—Creolin. By Dr. Wm. H. Potter. - - - 112

" IV.—Salol. By Alfred Eichler, M. D. - - - 118

" V.—The Rational Use of Vulcanizable Rubber. By W. N. Murphy, D. D. S. - - - - - 124

" VI.—Antiseptic Treatment of Wounds. By H. J. Warmuth, M. D. - - - - - - 128

" VII.—The Use of Cocaine as an Anæsthetic. - - 132

" VIII.—Safety of Hypnotism as a Remedy. - - - 134

### EDITORIAL.

Philosophy of Nutrition, with its Legal Aspect. - - - 136

Dr. Slich's Discovery. - - - - - - - 139

### MONTHLY SUMMARY.

Permanent Antiseptic Irrigation. - - - - - - 139

Manipulative Treatment and Dental Pathology - - - 141

Music in the Treatment of Nervous Diseases. - - - - 143

Trigeminal Neuralgia and Iodide of Potassium. - - - 144

